# An Interesting Case

**Published:** 1895-10

**Authors:** William J. Waugh

**Affiliations:** Third Cavalry, U. S. A.


### AN INTERESTING CASE.

BY WILLIAM J. WAUGH, V.S.,
THIRD CAVALRY, U. S. A.

A NON-GRADUATE veterinary practitioner called me in consultation early last summer to see a very sick Durham cow at Reno, Oklahoma Territory. The subject puzzled the attendant and the owner, hence they sought my assistance.

The patient was a large and valuable milch-cow that had been imported into the Territory, and was allowed to pasture at large on the public commons, where she had picked up and attempted to eat and swallow a large piece of cloth—which had been used as a kitchen dish-rag—and this had lodged as a foreign body in the œsophagus. She was considerably bloated and had been tapped several times with trocar and canula; but this afforded only temporary relief. I diagnosed the trouble as œsophageal obstruction in the thoracic region. We cast and secured her, as she was inclined to be vicious; then applied a mouth-gag or speculum, introduced a celluloid probang which encountered the obstruction in the thoracic portion of the œsophagus, and it was easily forced down into the rumen; but when I attempted to withdraw the instrument I found it had broken off at the joint, and the lower half remained in the animal. It was now late in the evening, and we released the cow, and she drank three bucketsful of cold water, and I informed the owner that I would return in the morning and operate to remove the instrument.

We cast and secured the cow in the usual way and made an incision on left side over rumen, inserted large needle and strong twine, and made a loop-stitch through rumen on each side of the point where I intended making an incision, then withdrew portion of rumen and made an incision about five inches, introduced my hand into the rumen and passed it along toward the œsophagus and found broken portion of probang in rumen and partly in œsophagus, then pushed it upward and withdrew it through the incision without disturbing the contents of the rumen, except removing the troublesome dish-cloth. I sutured the wound in rumen and washed cleanly with crude creosote lotion, then sutured the abdominal wound, leaving considerable opening at lower part for drainage; dressed with boiled linseed and crude creosote—seven of the former to one of the latter. The breath emitted a very fetid odor for about ten days, but we were unable to determine whether this was caused by ulceration of the œsophagus, due to pressure of foreign body, or from wound in rumen. The external wound discharged considerable pus, but there was no unfavorable results; and the cow made an early recovery and continued a good milker and breeder.

Crude creosote mixed with tallow is used extensively as an ointment to dress wounds and kill screw-worms among live-stock in the Southwest. However, some stockmen use crude creosote and pack the wound full of dried horse-feces, which proves an effectual and convenient treatment in that country.

Experience convinces me that it is never safe to use celluloid probangs in veterinary practice. A similar case in equine practice would probably have terminated fatally and resulted in litigation.

Dr. N. Recktenwald, of Pittsburg, Pa., has devised a very excellent probang, consisting of a small rubber-tube or hose filled with pliable rattan-rods and surmounted at ends with perforated brass caps fastened securely into the rubber-tubing or hose.

